# Aortic Isthmus Arteritis: Report of One Case

**DOI:** 10.4021/cr100e

**Published:** 2011-11-20

**Authors:** Ning Bao, Hui Ru Cao, Wen Hui Zhang, Yu Zhang

**Affiliations:** a Medical undergraduate, Jilin University, Changchun, Jilin province, China; b Department of Cardiology, the First Hospital of Jilin University, Changchun 130021, Jilin province, China

**Keywords:** Arteritis, Aortic isthmus, Hypertension, Aortic coarctation, Replacement

## Abstract

An 18-year-old girl was diagnosed as “bronchiectasis” for hemoptysis and treated by using embolization intervention 19 months ago. Two months ago she was diagnosed as iron-deficiency anaemia for fatigue. Eight days ago she was diagnosed again as hypertension for headache, anxiety, frowsty, nausea, vomiting and blood pressure 180/70 mmHg. In order to know the etiology of hypertension she was sent to our hospital. Vascular murmur was heard in bilateral carotid, subclavian and the back. 4 / 6 pan systolic murmur and stronger heart sound were heard in each valve auscultation area. Bilateral radial artery pulsations were symmetrical, but bilateral femoral, popliteal and dorsal arteries of foot were weakened. The results of hemoglobin (HB), globulin, creaction protein (CRP) and erythrocyte sedimentation rate (ESR) were abnormal. Thicker wall and narrower lumen in decreasing aorta were found by aorta CTA scanning. The aorta arteritist was clearly diagnosed and treated by hormone until ESR returned to normal. Finally, artificial vascular was replaced successfully by surgery. Now the patient is fine and has already been working for a year. This case gives us the inspiration: A detailed examination to patient is very important, which avoid missed diagnosis or misdiagnosis and missed the best opportunity for treatment.

## Introduction

Arteritis is a chronic non-specific inflammation involving the aorta and its main branches, which lead to vascular stenosis or occlusion. It is also called as Takayasu's arteritis which was first described by Takayasu in 1908. The systemic inflammatory response and organ ischemia are the main clinical manifestations. Compared with coronary atherosclerosis, hypertension and other common cardiovascular disease, arteritis is relatively rare disease. Etiology and pathogenesis is not clear yet. Because of a variety of clinical manifestations, there is a little difficulty in early diagnosis and treatment with little experience [1]. Report is as follows.

## Case Report

An 18-year-old girl was diagnosed as “bronchiectasis” for hemoptysis and treated by using embolization intervention 19 months ago. Two months ago she was diagnosed as iron-deficiency anaemia for fatigue. Eight days ago she was diagnosed again as hypertension for headache, anxiety, frowsty, nausea, vomiting and blood pressure 180/70 mmHg. In order to know the etiology of hypertension she was sent to our hospital. Physical examination shown: Right arm blood pressure was 180/70 mmHg and left arm 165/60 mmHg. Double lower limb blood pressure was not measured. Vascular murmur was heard in bilateral carotid, subclavian and the back, however, not in abdominal. 4/6 pan systolic murmur and stronger heart sound were heard in each valve auscultation area. Bilateral radial pulses were symmetrical, but bilateral femoral, popliteal and dorsal artery of foot was weakened. Laboratory tests were HB 9.5 g/L, globulin 38.4 mmol/L, CRP 29 mg/L and ESR 55 mm/h. Heart color Doppler showed increase of left ventricular diameter and left ventricular systolic function impairment. Vascular ultrasound showed decrease of blood flow velocity in double lower limb arteries, but normal in bilateral carotid, subclavian. At last, we gave her chest aorta CTA scanning, which thicker wall and narrower lumen in decreasing aorta were found.

According to above, the patient was diagnosed with Takayasu arteritis. Anti-inflammatory, hormone, immunosuppressive agents and antihypertensive drugs etc were used until ESR returned to normal. Finally, artificial vascular was successfully replaced by surgery. Now the patient is Ok and has already been working for a year. (Fig. A, B)

**Figure 1:**
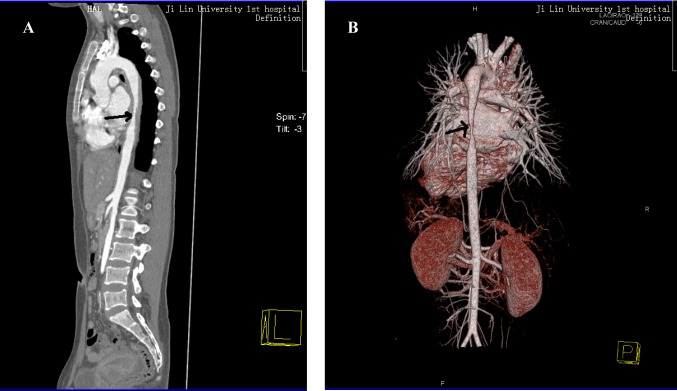
(A) lateral; (B) positive: After the contrast agent was injected by intravenously, thicker wall and narrower lumen were demonstrated in the distal aortic arch and part of the descending aorta. The smallest lumen was about 0.45 cm.

## Discussion

According to diagnostic criteria formulated in 1990 by the American rheumatism association the patient was clearly diagnosized as the aorta arteritis. Sunamori et al discovered through autopsy that all of the patients with arteritis were accompanied by different degree change of pulmonary [2, 3]. Haemoptysis might be considered as the earliest symptoms of arteritis. So, in order to diagnose early we should make examination of CTA to the patient with haemoptysis, especially young women. In this patient there was no enough evidence of involving the pulmonary artery, we do not discuss here. Aortic arch and its branch artery or abdominal aorta and renal artery were often involved in 84% patients with arteritis [2]. But aortic isthmus involvement is rare. We should pay more attention to identify with the congenital coarctation of aorta, which was considered as follows: 1) More incidence in men than women 3-5:1. 2) Thoracic-descending aorta more involved, the aorta spondylolysis in infantile and artery catheter junction in adult. 3) Specific and limited stenosis found by angiography. 4) Without systemic disease activity and abdominal murmurs [4].

However, characteristics in this patient were as fellows: 1) Increase of ESR and CRP indicated the infective diseases as well as its activity. 2) Vascular murmur represented stenosis of vascular lumen. 3) Thickening arterial wall also clew arteritis. 4) One of arteritis etiology was known as estrogen disorder in which lead to abnormal menstruation and hematopoietic inhibition. One-third of patients with arteritis are accompanied by anemia [5]. 5) Shortage of abdominal and lower limb blood supply caused reflexively the high blood pressure which lead to left ventricular enlargement and cardiac dysfunction finally. Therefore, the patient was diagnosed with Takayasu’s arteritis, instead of the congenital coarctation of the aorta. This kind of arteritis is suitable for surgery.

This case gives us inspiration: A detailed examination to patient is very important, which can avoid missed diagnosis or misdiagnosis or missed the best opportunity for treatment. If the patient had been diagnosed correctly in 19 months ago or 2 months ago, she would not have suffered later body pain. If this had still not been diagnosed as arteritis, the patient could have beard the onset of complications caused by poor control of hypertension, which leads to disability or death. This would be a very bad result to patient and her family. If the patient was diagnosed by other hospital later, we would not only lose credibility, but also face the medical disputes. So, as a doctor, we must have solid basic skills and do not underestimate the role of stethoscope in any time.

## References

[1] Feng Tian-jie, Wang Zeng-wu, Zhang Yi (2008). Diagnosis and treatment of Takayasu arteritis. Chinese Journal of hypertension.

[2] Ye Ren-gao, Lu Zai-ying, Xie Yi, Wang Chen (2004). The sixth edition of internal.

[3] Sunamori M, Hatano R, Yamada T, Tsukuura T, Sakamoto T (1976). Aortitis syndrome due to Takayasu's disease. A guideline for the surgical indication. J Cardiovasc Surg (Torino).

[4] Wei Meng (2004). The diagnosis and differential diagnosis of cardiovascular diseases.

[5] Jiang Jian-jun (2001). Peripheral vascular disease.

